# Allosteric Antagonism of Insect Odorant Receptor Ion Channels

**DOI:** 10.1371/journal.pone.0030304

**Published:** 2012-01-17

**Authors:** Patrick L. Jones, Gregory M. Pask, Ian M. Romaine, Robert W. Taylor, Paul R. Reid, Alex G. Waterson, Gary A. Sulikowski, Laurence J. Zwiebel

**Affiliations:** 1 Department of Biological Sciences, Vanderbilt University, Nashville, Tennessee, United States of America; 2 Department of Chemistry, Vanderbilt Institute of Chemical Biology, Vanderbilt University, Nashville, Tennessee, United States of America; 3 Department of Pharmacology, Vanderbilt University Medical Center, Nashville, Tennessee, United States of America; 4 Center for Molecular Neuroscience, Institute of Global Health and Program in Developmental Biology, Vanderbilt University Medical Center, Nashville, Tennessee, United States of America; University of Arizona, United States of America

## Abstract

**Background:**

At a molecular level, insects utilize members of several highly divergent and unrelated families of cell-surface chemosensory receptors for detection of volatile odorants. Most odors are detected via a family of odorant receptors (ORs), which form heteromeric complexes consisting of a well-conserved OR co-receptor (Orco) ion channel and a non-conserved tuning OR that provides coding specificity to each complex. Orco functions as a non-selective cation channel and is expressed in the majority of olfactory receptor neurons (ORNs). As the destructive behaviors of many insects are principally driven by olfaction, Orco represents a novel target for behavior-based control strategies. While many natural and synthetic odorants have been shown to agonize Orco/Or complexes, only a single direct Orco modulator, VUAA1, has been described. In an effort to identify additional Orco modulators, we have investigated the structure/activity relationships around VUAA1.

**Results:**

A search of our compound library identified several VUAA1 analogs that were selected for evaluation against HEK cells expressing Orco from the malaria vector *Anopheles gambiae* (*AgOrco*). While the majority of compounds displayed no activity, many of these analogs possess no intrinsic efficacy, but instead, act as competitive VUAA1 antagonists. Using calcium mobilization assays, patch clamp electrophysiology, and single sensillum *in vivo* recording, we demonstrate that one such candidate, VU0183254, is a specific allosteric modulator of OR signaling, capable of broadly inhibiting odor-mediated OR complex activation.

**Conclusions:**

We have described and characterized the first Orco antagonist, that is capable of non-competitively inhibiting odorant-evoked activation of OR complexes, thereby providing additional insight into the structure/function of this unique family of ligand-gated ion channels. While Orco antagonists are likely to have limited utility in insect control programs, they represent important pharmacological tools that will facilitate the investigation of the molecular mechanisms underlying insect olfactory signal transduction.

## Introduction

Insect behavior is largely directed by the sensation of environmental olfactory cues [1]. More important to human health, the destructive behaviors of disease vector mosquitoes and related dipterans are driven by the sensory modality of olfaction, making it an important area of study [2]. AgOrs and other insect ORs belong to a large and highly divergent superfamily, capable of discerning a broad range of chemical odorants [3]. The breadth and size of the OR family varies between insects, where these traits combine to generate a remarkably diverse chemosensory repertoire [4]. Individual tuning AgORs are functionally defined by their responses to various odorants, and these responses can vary widely [5], [6]. The OR co-receptor (Orco) is required for all OR-based chemoreception in insects, which is the only lineage to possess this unique and highly conserved ion channel that is present in most ORNs [7], [8], [9]. Insect ORs are distinct from their mammalian counterparts in that they are not related to any known GPCRs and possess an inverse 7-TM topology [10], [11]. Recently it was shown that Orco is a non-selective cation channel, but it is unclear what roles, if any, second messengers may play [12], [13], [14], [15]. In heterologous expression, Orco is capable of forming functional channels independent of any tuning OR, although the *in vivo* consequence of this capacity is unknown [14]. Tuning ORs expressed in the absence of Orco have no demonstrable functional capacity in heterologous systems or *in vivo,* as Orco is required not only for proper signal transduction, but also for trafficking of the OR complex to the ORN membrane [7], [8], [10].

Classically, insect tuning ORs have been defined by their ability to respond to various odorants, but always in complex with Orco. In contrast, Orco does not have a defined natural ligand, and as a result, its direct study has until recently not been possible. The first Orco agonist, VUAA1, is a synthetic molecule that was discovered in a chemical screen designed to identify AgOR modulators for insect control with demonstrable activity *in vivo* [14]. In a continuation of these studies, we sought to identify related compounds in order to uncover more diversity within this novel class of Orco modulators.

## Results and Discussion

To better define the structure/activity relationships (SAR) of VUAA1 analogs we performed calcium mobilization assays in HEK cells expressing AgORs. While examining compounds in the Vanderbilt Institute of Chemical Biology (VICB) collection that shared structural similarities with VUAA1, a large number of compounds were observed that possessed no intrinsic agonist activity (data not shown). As a number of these compounds were structurally related to VUAA1, we hypothesized that a subset of these analogs may nevertheless have affinity for Orco, but lack efficacy, and thus would be classically described as antagonists.

To investigate these analogs as VUAA1 antagonists, we examined whether they were capable of suppressing VUAA1-mediated responses of AgOrco-expressing HEK cells in calcium mobilization assays [14]. In these competition experiments, we identified a number of putative antagonists for further study (data not shown) and chose to pursue two antagonists for further study based on their differing efficacies and structural characteristics. The first antagonist, VU0450667 is almost structurally identical to VUAA1, possessing the same triazolopyridine core as VUAA1 and differing only in the pattern of aniline substitution. A related analog, VU0183254, possesses a furanotriazole core appended to a phenothiazine. It is interesting to note that despite their similarity to VUAA1, they possessed no agonist activity, demonstrating a narrow structure/activity relationship of VUAA1-mediated Orco agonism.

**Figure 1:**
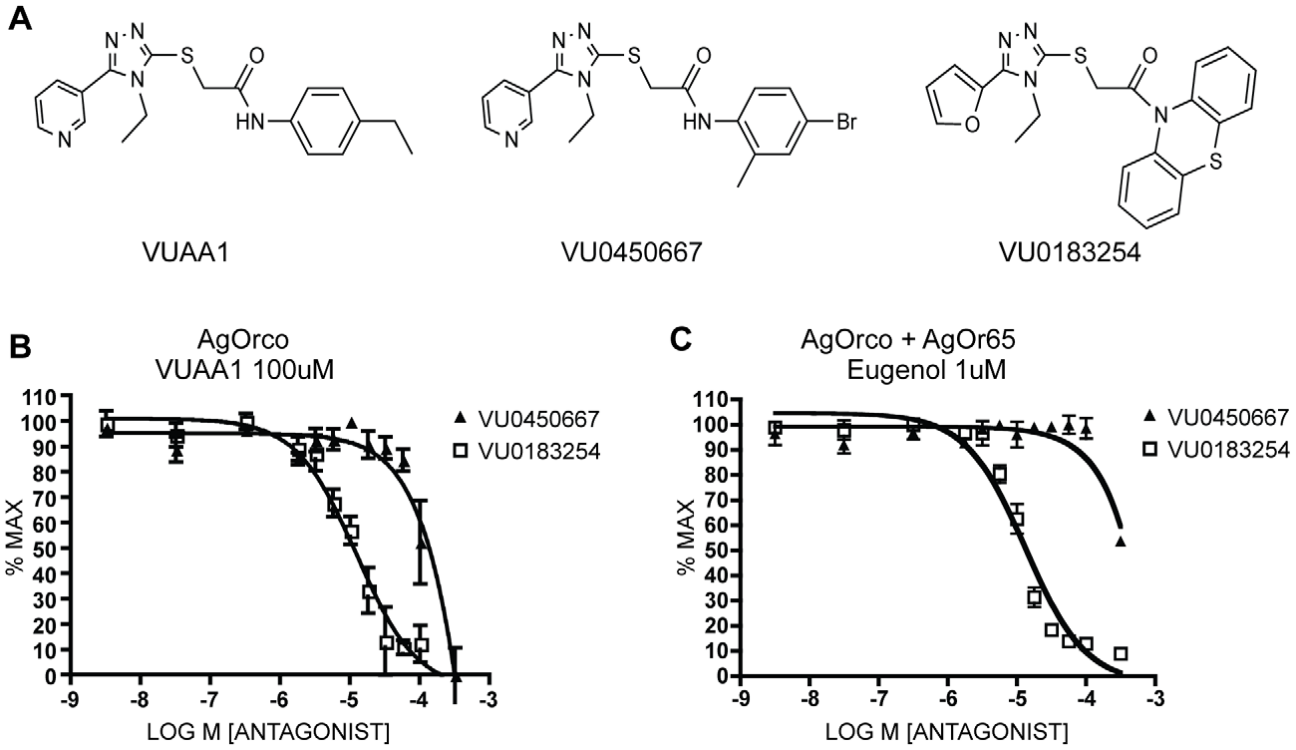
VUAA1 analogs can antagonize AgOr responses. **A.** Structures of the Orco agonist VUAA1, Orco antagonist VU0183254, and control VU0450667. **B.** IC_50_ curve of the response curves of AgOrco-only expressing HEK cells in the presence of pre-loaded antagonist (VU0183254 or VU0450667) followed by an addition of VUAA1 to 100 µM; measurements were taken on an FDS6000 using Fluo4-AM as described in methods **C.** IC_50_ curve of AgOrco+AgOr65 expressing HEK cells as in **B.**, followed by addition of eugenol to 1 µM.

When AgOrco was pre-treated with either of the two related antagonists, VU0183254 or VU0450667, before VUAA1 addition, they both strongly inhibited VUAA1 activation of Orco (Figure 1A,B). The IC_50_ values were −4.9+/−0.09 and −2.8+/−0.72 log M (+/−SEM) for VU0183254 and VU0450667 respectively. We next investigated whether these VUAA1 analogs were capable of antagonizing odor-mediated activation of the Orco complex in the same manner as above. Initially, to investigate the potential for non-competitive antagonism of an Orco-Or complex, we chose AgOR65 as the tuning OR, which is potently agonized by eugenol [5], [6]. Compound VU0183254 strongly antagonized odorant-mediated activation of AgOrco-AgOr65 (IC_50_ −4.8+/−0.06 (LogM+/−SEM)) (Figure 1C). Interestingly, although the 4-bromo-2-methylaniline-based amide analog of VUAA1 (VU0450667) acts as an antagonist of VUAA1-mediated complex activation, it was virtually incapable of inhibiting odor-mediated activation. This was unexpected and suggests these two molecules may be antagonists that act via differing mechanisms.

**Figure 2:**
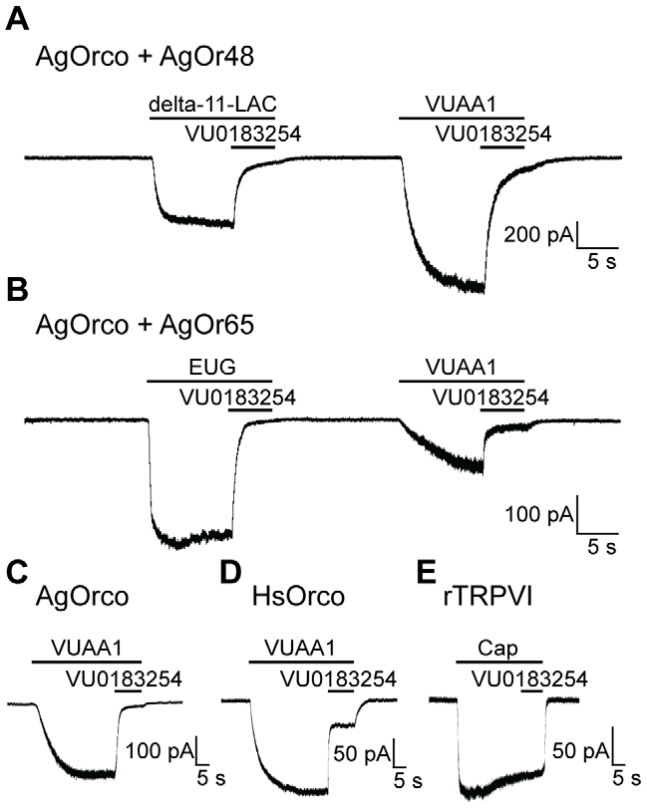
VU0183254 reduces OR-mediated currents. **A**–**B**. Whole-cell patch clamp recordings of odorant- and VUAA1-induced currents in AgOr-expressing cells (n = 3). **A.** VU0183254 (100 µM) decreased responses to delta-undecalactone (1 µM) and VUAA1 (100 µM) in HEK cells expressing AgOrco+AgOr48. **B.** Responses to eugenol (1 µM) and VUAA1 (100 µM) in AgOrco+AgOr65 cells were reduced by VU0183254 (100 µM). **C**–**D**. Cells expressing Orco from either *An. gambiae* (**C**) or *Harpegnathos saltator* (**D**) had reduced VUAA1 (100 µM) currents in the presence of 100 µM VU0183254. **E.** Capsaicin (10 µM) currents in HEK cells expressing rat TRPVI were not reduced with by 100 µM VU0183254. Holding potential for all figures is −60 mV.

To further examine the antagonist properties of VU0183254, we tested AgOR complexes in HEK cells using whole-cell patch clamp electrophysiology. When AgOrco+AgOr48 was treated with the strong ligand delta-undecalactone, large macroscopic currents were evoked that were reduced by 85.1±1.8% in the presence of VU0183254 treatment (Figure 2A). VUAA1-evoked currents of AgOrco-AgOr48 cells were similarly reduced (85.5±1.5%) after VU0183254 treatment (Figure 2A). Another AgOR complex, AgOrco+AgOr65, was also highly susceptible to VU0183254 current reduction in the presence of both eugenol (89.0±4.8%) and VUAA1 (85.0±3.5%) (Figure 2B). These results validate and extend the calcium mobilization concentration response curve (CRC) studies as well as demonstrate that VU0183254 is a potent antagonist of both VUAA1 and odor-mediated activation.

We next investigated whether VU0183254 is capable of acting as a universal Orco antagonist or whether it is specific to AgOrco by performing whole-cell patch clamp experiments in HEK cells that transiently express Orco channels from the ponerine ant, *Harpegnathos saltator.* When HsOrco, which is 62% identical to AgOrco, was activated with VUAA1, VU0183254 reduced VUAA1-mediated macroscopic currents by 63.9±2.2%. In these assays, VU0183254 showed even stronger inhibition of VUAA1 currents (89.5±7.5%) from AgOrco-only expressing cells (Figure 2C,D). These differences suggest that, despite the conservation of Orco across insects, the potency of VU0183254 can vary across orthologous Orco channels. To test for specificity of VU0183254 action, we examined its effect on the *Rattus norvegicus* TRPV1 receptor, a nonselective cation channel unrelated to the OR family [16]. Here, VU0183254 did not affect the macroscopic currents induced by the natural TRPV1 ligand capsaicin, although we note the natural desensitization of the receptor in the presence of the agonist (Figure 2E). These results demonstrate that VU0183254 does not act as a broad-spectrum cation channel antagonist, although we cannot rule out effects on other channel families beyond the scope of this study.

**Figure 3:**
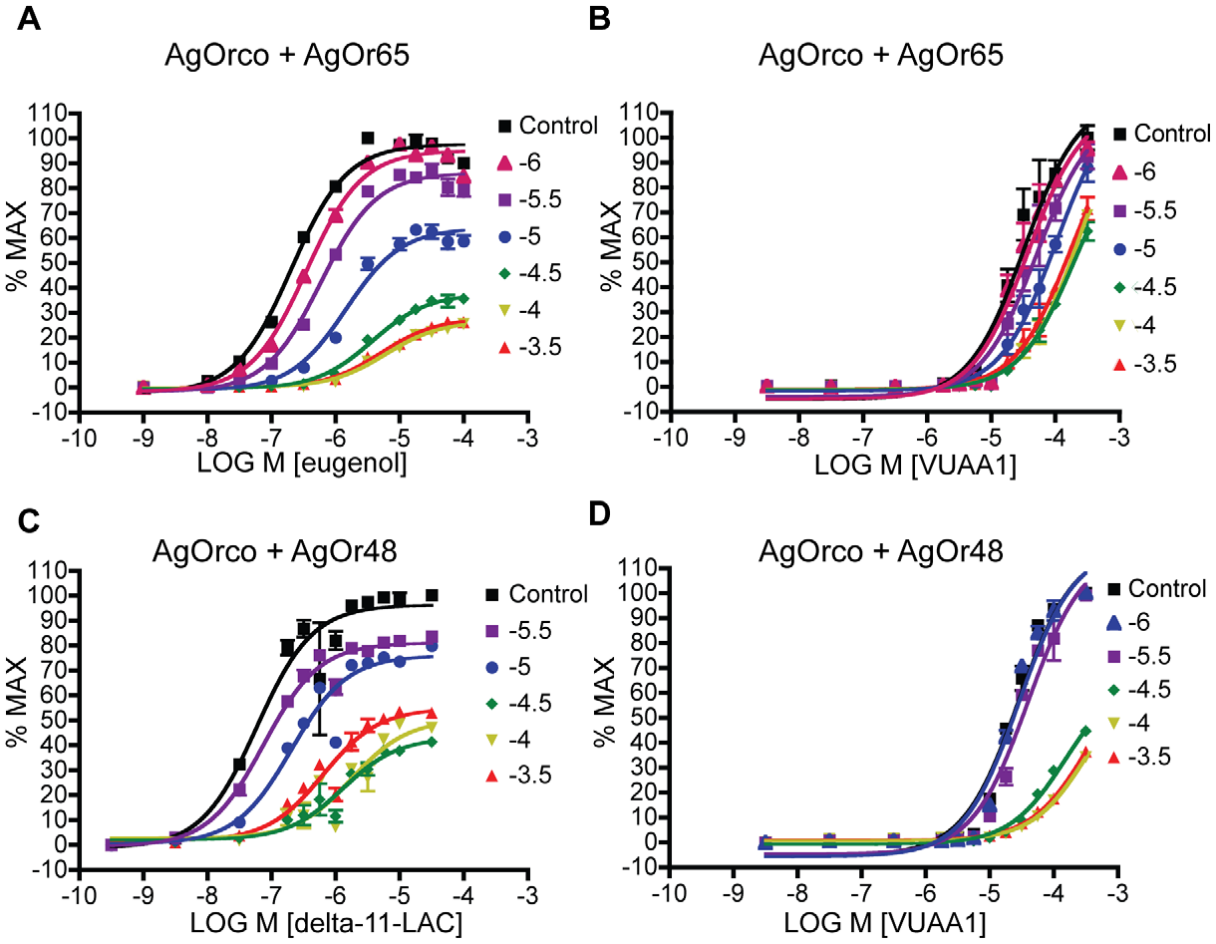
VU0183254 is an allosteric antagonist. **A.** Concentration response curves of AgOrco+AgOr65 expressing HEK cells in the presence of a series of steady concentrations (expressed as logM) of pre-loaded VU0183254 (different colored lines, see inset) followed by increasing concentrations of eugenol as measured using Fluo-4AM and an FDSS6000. **B.** As in A with VUAA1. **C.** As in A, with AgOrco+AgOr48 and delta-undecalactone. **D.** As in C with VUAA1. In all cases results are shown as means+/−SEM, n = 4.

To determine the pharmacological nature of VU0183254-mediated antagonism of both VUAA1 and odorant stimulation, we examined the effect of VU0183254 treatment on AgOrco in complex with the two odorant tuning AgOrs used above, AgOr48 and AgOr65. These tuning ORs are both narrowly tuned and are 15% identical, yet do not share any functional overlap [5], [6]. These ORs were chosen because of their disparate identities and ligand specificities. In addition, the OR's respective ligands elicit responses at low nanomolar concentrations allowing CRCs to be examined across a full range of responses. The effect of VU0183254 was determined by pre-treating OR complexes with antagonist, followed by agonist addition, such that multiple agonist CRC's were run in the presence of increasing concentrations of pre-treated antagonist. When AgOrco+AgOr65 cells were pre-treated with antagonist and then treated with eugenol, maximal responses were depressed 70% compared to control. In addition, curves were shifted dextrally as antagonist concentration was increased (Figure 3A). In the absence of antagonist, the EC_50_ of eugenol was found to be −6.69+/−.03 (LOGM+/−SEM), whereas in the presence of −3.5LogM VU0183254, the EC_50_ was shifted to −5.286+/−.04(LOGM+/−SEM). Importantly, these studies show the antagonism of VU0183254 was insurmountable; increasing concentrations of odorant agonist were incapable of producing a maximal response equivalent to the no-antagonist control. Furthermore, VU0183254 displayed insurmountable antagonism of AgOrco+AgOr48 stimulation by delta-undecalactone (Figure 3C). These results are consistent with the conclusion that VU0183254 acts as a broad-spectrum, non-competitive antagonist against odorant-induced activation of AgOrco+AgOr complexes.

It is important to note that for both receptor complexes, antagonism becomes limited at −100 µM. Despite increasing concentrations of antagonist, further depressions in signal are not seen. The mechanism of complete and insurmountable antagonism, such as was seen with odor-mediated antagonism of AgOrco+AgOr65 and AgOrco+AgOr48, is non-competitive. However, to define an insurmountable antagonist as allosteric, it must be distinguished from a non-competitive orthosteric antagonist [17]. When allosteric antagonists saturate a receptor site, their antagonism becomes limited, such that further effects cannot be elicited despite increasing concentrations of antagonist [17]. The inhibition of odor-mediated activation of AgOrco+AgOr complexes by the VUAA1 analog VU0183254 is therefore consistent with limited insurmountable antagonism, and is thus classified as an allosteric antagonist.

We next examined the nature of VU0183254 antagonism of VUAA1-mediated agonism. When tested as above, VU0183254 was also shown to depress VUAA1-mediated agonism of AgOrco+AgOr48 or AgOr65 cells (Figure 3B, D). Here, however, equimolar antagonist concentrations were not nearly as effective in depressing VUAA1 responses as they were odorant responses. The insolubility of VUAA1 in assay buffer at concentrations greater than 100 µM prevented testing at the concentrations required to assess whether this antagonism was surmountable. However, the general trend within this data is a dextral curve shift as antagonist concentration increases, consistent with a competitive mode of antagonism. This data, in addition to the close structural relationship of VU0183254 and VUAA1, further supports a competitive mode of action for this analog insofar as VUAA1 is concerned. Taken together, these results provide strong support for the conclusion that VU0183254 acts a competitive antagonist of VUAA1, but is a non-competitive, allosteric antagonist of odor-mediated activation of OR complexes. This conclusion is further supported by the data shown in Figure 1, in which VU0450667 was largely incapable of inhibiting odor-mediated activation, but VU0183254 was, whereas both were capable of inhibiting VUAA1 activation. This probe dependence is another indicator of allosteric modulation.

In order to assess the effect of VU0183254 on Orco-expressing ORNs *in vivo*, we performed passive electrophysiological recordings on single capitate peg (Cp) sensilla of adult female mosquitoes. Cp sensilla are found on the maxillary palps of *An. gambiae* and are innervated by three olfactory receptor neurons in a highly stereotyped pattern: CpB and CpC express Orco, while CpA is narrowly tuned to CO_2_ and does not express Orco [18]. Observing the activity of these three neurons in a single-sensillum recording (SSR) is accomplished by puncturing the sensillum with a pulled-glass electrode and collectively sampling the activity of CpA, B, and C neurons simultaneously. CpA spike activity can be distinguished by its larger amplitude and its response to CO_2_ while CpB and CpC spikes cannot be readily distinguished from each other, and are consequently binned during analysis.

Previously, an increase in CpB/C activity was observed upon treatment of Cp SSR preparations with the Orco agonist VUAA1 [14]. Based on its Orco antagonist activity, we introduced VU0183254 to Cp sensilla via the recording pipette with the expectation that the presence of this compound would reduce the spontaneous firing rate of AgOrco-expressing CpB/C neurons without affecting the activity of CpA. We based this expectation on previous results demonstrating that in SSR assays using *DmOrco^−/−^* mutants of *Drosophila melanogaster,* ORN spontaneous activity is significantly reduced [19]. In addition, exogenous expression of different ORs in the *Drosophila* empty-neuron system, which is DmOrco positive, but lacks other tuning ORs, results in varying spontaneous activities [8]. Together, these data suggest that the OR complex is responsible for determining the spontaneous activity of ORNs and that an Orco antagonist would thus be expected to reduce the spontaneous activity of these Orco-containing neurons.

**Figure 4:**
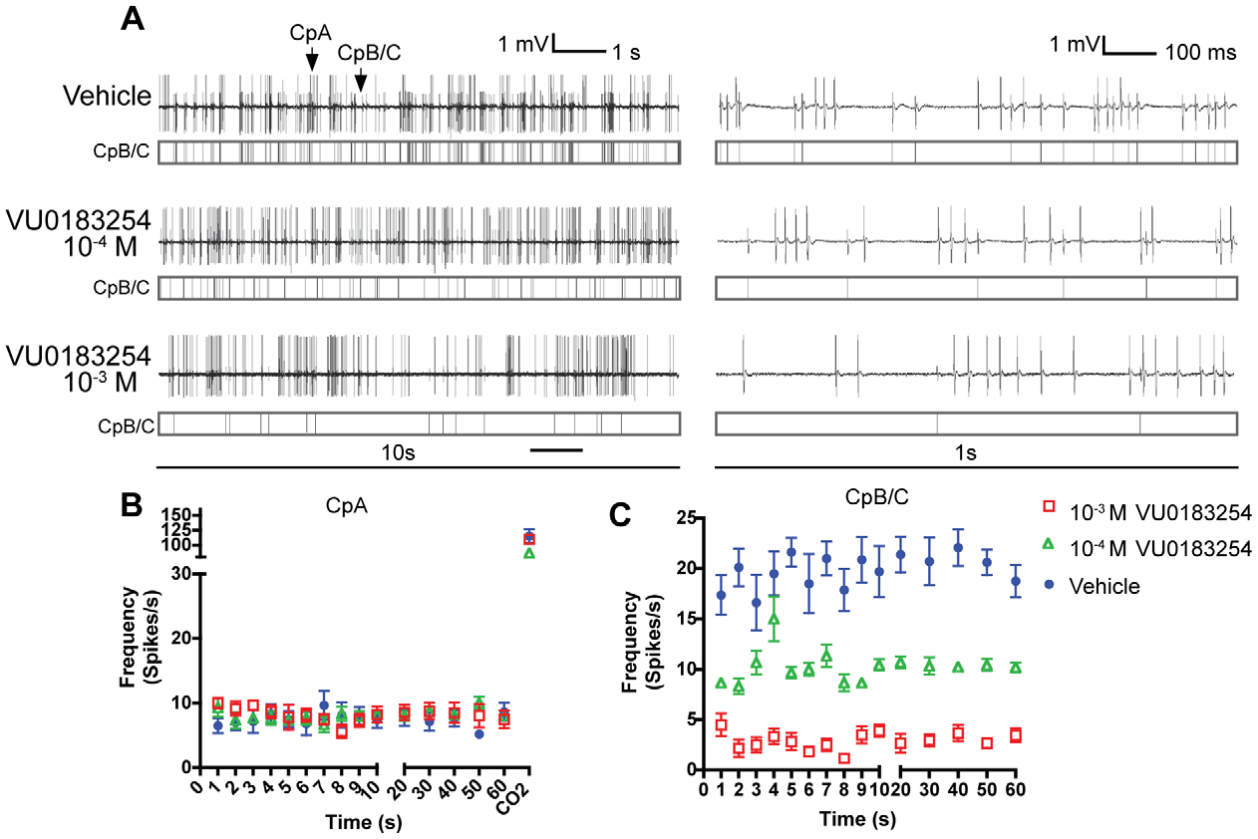
VU0183254 reduces Orco-mediated activity *in vivo.* **A.** Representative traces of Cp neuron activity in response to vehicle (DMSO) or VU0183254 as measured by single-sensillum electrophysiology. Activity was allowed to stabilize for 10 seconds and then recorded for 60 seconds. CpA spikes can be distinguished by their larger amplitude in expansions (right panel). CpB/C spikes (counts below traces) are reduced in the presence of VU0183254. **B.** Spontaneous CpA spike rates are unaffected by the presence of VU0183254 (n = 8). Spikes were counted for each of the first 10 seconds and then averaged across the remaining 10-second intervals. Normal CpA activity is confirmed by CO_2_ pulse delivered at the end of the recording period. **C.** Spontaneous firing rates of CpB/C neurons are reduced by VU0183254 in a dose-dependent manner (n = 8). All spikes were distinguished and quantified using AutoSpike software (Syntech).

Palpal SSR studies were carried out in *An. gambiae* such that spike activities were allowed to stabilize for 10 seconds in CO_2_–free air following sensilla puncture, and then recorded for a period of 60 seconds, followed by a pulse of CO_2_ to confirm CpA sensitivity. Delivery of VU0183254 to treated Cp sensilla had no effect on the spontaneous or CO_2_-evoked spike frequencies of the CpA neuron (Figure 4A, B), but was indeed able to reduce the spontaneous firing rate of CpB/C neurons in a dose-dependent manner (Figure 4A, C). This finding strongly suggests that VU0183254 is able to specifically antagonize spontaneous AgOrco-mediated neuronal activity *in vivo*.

**Figure 5:**
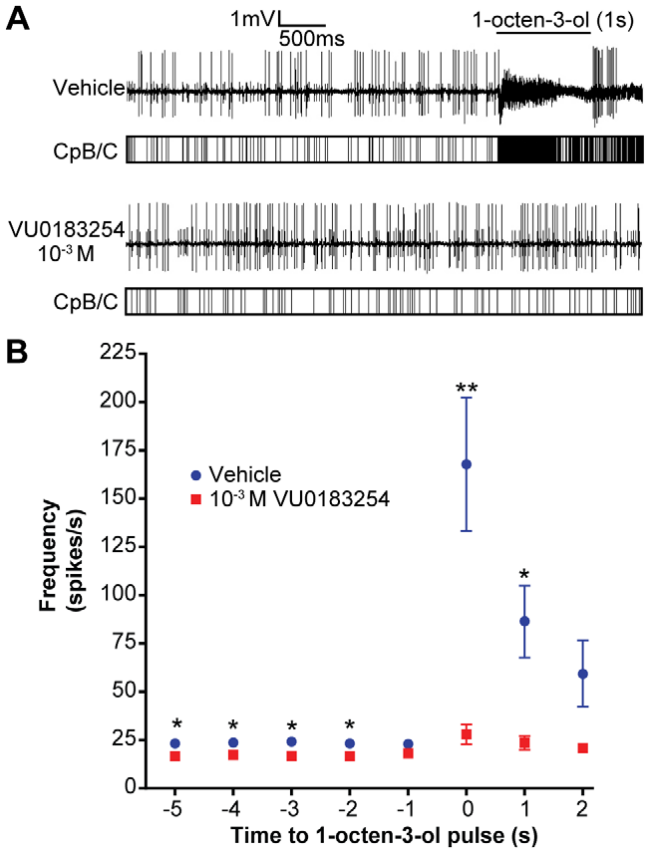
VU0183254 antagonizes odor-evoked ORN activity *in vivo*. **A.** Representative traces of Cp neuron activity in response to a 1 s pulse of 1-octen-3-ol (dark bar above traces). Vertical lines under each raw trace indicates spike counter for CpB and CpC spikes only. Vehicle-only trace (top) responds strongly to 1-octen-3-ol, while preparations exposed to VU0183254 (bottom) do not respond. **B.** Quantification of traces as in (A). Spike frequencies were binned every second for 5 seconds prior to 1-octen-3-ol pulse and for 2 seconds afterwards. In the presence of vehicle alone (blue circles, n = 11) CpB/C neurons respond to a 1 s pulse of 1-octen-3-ol with increased activity, while preparations including VU0183254 (red squares)(n = 11) do not respond as strongly (*, p<0.05; **, p<0.005).

If VU0183254 is able to reduce the spontaneous activity of CpB/C neurons by antagonizing Orco, we hypothesized that this compound may also be able to antagonize odorant-mediated activation of Orco-expressing ORNs in the Cp sensilla preparation. Indeed, CpB/C neurons exposed to recording solution with solvent alone are strongly activated by the volatile odorant 1-octen-3-ol (167.8+/−34.1 spikes/s, N = 11) (Figure 5A), but this activation is strongly inhibited by the presence of VU0183254 (28.1+/−4.98 spikes/s, N = 11, p = 0.0026) (Figure 5B). Taken together, these results demonstrate that VU0183254 is capable of suppressing passive ORN spontaneous activity as well as odor-evoked activity. These studies further implicate AgOrco as the principal facilitator of ORN spike activity.

### Conclusion

These studies further our understanding of the nature of insect OR-based signaling by demonstrating the existence of a universal Orco–specific antagonist, which acts independently of odorant stimulation. Because of Orco's novelty relative to other ligand-gated channels, and until recently the inability to study it directly in the absence of an odorant-specific tuning OR, little is known about its gating mechanisms. The identification of the VU0183254 antagonist provides new insight, in that it possesses affinity for the Orco channel, most likely in the closed gating state. We suggest that VU0183254 allosterically “locks” the channel shut, preventing odorants from orthosterically activating the channel complex.

Despite their potential utility in the study of insect OR signaling mechanisms, there is little justification for the development of Orco antagonists as insect control agents. The ability of Orco or tuning OR antagonists to act as behavioral “confusants” is compromised because, unlike laboratory studies involving unitary odorant stimuli, most host/target organisms emit complex blends of multiple semiochemicals that act as kairomones. Discrete components of these chemical blends as well as other host-derived stimuli are sensed in insects by ORs as well as OR-independent signaling pathways that are mediated by other large families of multi-modal cell-surface receptors [20], [21], [22] that are unlikely to be affected by Orco/OR antagonists. In contrast, broad-spectrum Orco agonists such as VUAA1 provide a more likely basis for the development of a new generation of insect repellents, as they are expected to act as excito-repellents that modulate insect behavior by hyper-stimulation of sensory receptors resulting in aversive behavioral responses.

While the molecular targets and mechanisms of most insect repellents are not fully characterized, Citronella and DEET, two of the most prevalent commercial agents, have both been characterized as activators of AgOrco/OR complexes and chemosensory neurons in several species [23], [24], [25], [26], [27]. Indeed, recent studies in mosquitoes have demonstrated the utility of hyper-agonists that target CO_2_ receptors as effective repellents [28]. While agonism of chemosensory receptors likely represents a powerful insect control approach, DEET has also been implicated in antagonism of some OR complexes, which further confounds its mechanism of action [29], [30]. While the ultimate utility of Orco antagonists remains to be seen, their discovery and characterization will serve as the foundation for the development of new pharmacological tools that can be used to further interrogate the novel channel class that insect ORs represent.

## Methods

### Cell Culture and Antagonist Testing

Creation of AgOr48 and AgOr65 HEK293 T-Rex (Invitrogen) cell lines was carried out as described [14]. Calcium mobilization assays were carried out essentially as described using Fluo4AM (Invitrogen) chemistry and an FDSS6000 plate reader (Hammamatsu) [14], [31]. Briefly, assay compounds were diluted in DMSO to 100 µM and transferred to a 384-well, polypropylene Echo Qualified Microplate (Labcyte) before being dispensed into 384-well polypropylene destination plates (Greiner) using an ECHO555 (Labcyte). Compounds were diluted in assay buffer (20 mM HEPES, 1× Hanks Buffered Saline Solution) using a Multidrop Combi (Thermo). To allow for antagonist equilibration, antagonists were added to OR-expressing cells 100 s prior to agonist addition. Fluorescence readings were taken at a frequency of 1/s for the duration of each assay. Responses were quantified by the ratio of the maximum fluorescent reading of a well divided by the baseline reading of that well prior to antagonist addition.

#### Chemicals and VU0450667 synthesis

VU0183254,10-({[4-ethyl-5-(2-furyl)-4H-1,2,4-triazol-3-yl]thio}acetyl)-10H-phenothiazine was purchased from ChemBridge's rare chemical library. Delta-undecalactone and eugenol were purchased from Sigma-Aldrich. VU0450667, N-(4-bromo-2-methylphenyl)-2-((4-ethyl-5-(pyridin-3-yl)-4H-1,2,4-triazol-3-yl)thio)acetamide synthesis; To a solution of 4-bromo-2-methylaniline (40.1 mg, 0.22 mmol) in 1.5 mL of CH_2_Cl_2_ was added triethyl amine (30 mL, 0.22 mmol) and chloroacetyl chloride (17 mL, 0.22 mmol). After 2 h, the solution was concentrated then redissolved in 1.5 mL of acetonitrile. To this solution was added 4-ethyl-5-(pyridin-3-yl)-4H-1,2,4-triazole-3-thiol (30 mg, 0.15 mmol) and cesium carbonate (98 mg, 0.3 mmol) After 16 h the reaction was concentrated and the residue was purified by column chromatography with MeOH/CH_2_Cl_2_ (1∶4) to afford 31 mg (50%) of the desired product: ^1^H NMR (CDCl_3_) d 9.97 (s, 1H), 8.85 (s, 1H), 8.78 (d, J = 3.3 Hz, 1H), 7.95 (dt, J = 1.8, 7.9 Hz, 1H), 7.91 (d, J = 9.3 Hz, 1H), 7.48 (dd, J = 4.9, 7.8 Hz, 2H), 7.29 (m, 2H), 4.07 (s, 2H), 4.00 (dd, J = 7.3, 14.5 Hz, 2H), 2.33 (s, 3H), 1.39 (t, 7.3 Hz, 3H); LRMS calculated for C_18_H_18_BrN_5_OS (M+H)^+^ *m/z*: 432.0 Measured 432.0 m/z.

#### Single Sensillum Recording

Recordings from 5–6 day-old female *An. gambiae* mosquitoes were performed as described previously ([14]). Statistical analysis consisted of Student's t-tests performed using Prism software (Graphpad).
